# Demoralization and Its Relationship with Depression and Hopelessness in Suicidal Patients Attending an Emergency Department

**DOI:** 10.3390/ijerph17072232

**Published:** 2020-03-26

**Authors:** Alessandra Costanza, Marc Baertschi, Hélène Richard-Lepouriel, Kerstin Weber, Isabella Berardelli, Maurizio Pompili, Alessandra Canuto

**Affiliations:** 1 Department of Psychiatry, Faculty of Medicine, University of Geneva (UNIGE), 1211 Geneva, Switzerland; aless.canuto@gmail.com; 2 Department of Psychiatry, ASO Santi Antonio e Biagio e Cesare Arrigo Hospital, 15121 Alessandria, Italy; 3 Service of General Psychiatry and Psychotherapy, Nant Foundation, 1820 Montreux, Switzerland; marc.baertschi@nant.ch; 4 Service of Psychiatric Specialties, Department of Psychiatry, University Hospitals of Geneva, 1211 Geneva, Switzerland; helene.richard-lepouriel@hcuge.ch; 5 Division of Institutional Measures, Medical Direction, University Hospitals of Geneva, 1211 Geneva, Switzerland; kerstin.weber@hcuge.ch; 6 Department of Neurosciences, Mental Health and Sensory Organs, Suicide Prevention Center, Sant’Andrea Hospital, Sapienza University of Rome, 00189 Rome, Italy; isabella.berardelli@uniroma1.it (I.B.); maurizio.pompili@uniroma1.it (M.P.)

**Keywords:** suicide, suicidal behavior, suicidal ideation, suicide attempt, demoralization, risk factor, emergency department

## Abstract

Emergency departments (EDs) play an increasingly crucial role in the management of patients with suicidal behavior (SB). Demoralization has been associated with SB in various populations and conditions, but little is known about the effect of this construct in SB patients who attend an ED. Therefore, a more inclusive SB assessment which considers the demoralization construct could be useful in clinical practice. The main aim of this study was to assess the presence and severity of demoralization in patients visiting EDs for SB. Secondly, the maintenance of the relationship between demoralization and SB after controlling for depression and the proportion of variance which accounted for hopelessness was investigated. A cross-sectional study of patients (*N* = 199) visiting an ED for SB was performed, which examined the role of demoralization, hopelessness, and depression on suicidal ideation (SI) and suicide attempts (SAs). Demoralization was strongly and positively correlated with SI. Demoralization was related to major depressive episodes, but it was confirmed to be a different and, probably, more sensitive construct for SB, validating its specificity in relation to depression. Hopelessness accounted for a small portion of the variance in SI, compared to demoralization. Formal support for the association of demoralization with SI was provided. Demoralization can improve SB assessment in EDs, particularly among patients whose suicide risk can be unnoticed. Furthermore, demoralization represents a clinically useful concept to increase comprehension of the suffering of the suicidal patient and a possible target for psychotherapeutic interventions.

## 1. Introduction

Emergency departments (EDs) represent the most privileged point of contact with the healthcare system, for patients presenting suicidal ideation (SI), or suicidal behavior (SB), in particular suicidal attempts (SAs) [1,2]. SI has been defined by Posner as “passive thoughts about wanting to be dead or active thoughts about killing oneself, not accompanied by preparatory behavior”, and SA as “a potentially self-injurious behavior, associated with at least some intent to die, as a result of the act” [3]. In addition, EDs have the potential to play a central role in suicide prevention, as individuals with SB discharged from EDs are at a high near-term risk of further SB [4,5,6,7]. Despite these advantages, EDs are underused by patients with SB, which may be attributed to the heterogeneity of the visiting population, e.g., somatic and/or psychiatric, social, or age-related issues, making SI and SA assessment difficult [1]. In addition, a diagnosis of depression or other psychiatric disorder is unclear in many cases, even for patients presenting with SI [1]. Approximately 8%–12% of patients visiting EDs for nonpsychiatric reasons do not disclose their SI [8,9]. This highlights the difficulties among patients and health professionals to identify or discuss suicidality. Accordingly, a proxy for SI and SA assessment, and more inclusive documentation of severe despair, potentially leading to SB, as well as consideration of the heterogeneous ED population, would be extremely valuable in improving the identification of individuals at risk of, or presenting with, SB.

A construct that can be associated with SB that has been described in various populations and conditions, including combat troops, holocaust survivors, immigrants, disadvantaged individuals, community samples, and patients with somatic or mental diseases, is demoralization [10,11]. It was first described by Frank [12] as a definite cluster of symptoms resulting from a “persistent failure to cope with internally or externally induced stresses that the person and those close to him expect him to handle. Its characteristic features, not all of which need to be present in any one person, are feelings of impotence, isolation, and despair” [12]. Demoralization was later conceptualized as a syndrome of distress combined with subjective incompetence, with the latter component constituting its clinical hallmark [13]. Through the integration of the giving up-given up complex [14], a set of criteria for the diagnosis of demoralization was proposed by Fava and colleagues [15], which examined this construct among groups of patients with different somatic disorders and integrated it into the psychosomatic domain [15,16,17,18]. Kissane and Clarke’s theoretical model proposed demoralization to be characterized by symptoms such as loss of meaning in life, hopelessness, helplessness, sense of failure, and dysphoria, focusing on terminally ill patients [19,20,21]. According to this model, existential distress is expressed across a spectrum of mental states; however, only its extreme form is potentially pathological, and a subset of the latter—characterized by the above-mentioned specific dimensions—configures the demoralization syndrome [20]. 

An association between demoralization and the potential development of SB was first proposed by Slavney in a study on patients referred from general medical and surgical areas; however, this author considered demoralization to be a normal response to adversity and not a condition characterized by specific pathological dimensions [22,23]. SB in demoralization was specifically addressed by Kissane, who approached the literature on demoralization because of a particular interest in suicide [24,25]. Kissane emphasizes the predictable progress of a desire to die or to commit suicide in patients under palliative care, who can be demoralized, but not necessarily depressed [19,20,26]. A progressive instillation of SB in demoralized patients was confirmed in the setting of somatic care, with or without a concomitant psychiatric disorder [18,27,28,29], in socially disadvantaged individuals [30] and schizophrenia [31,32]. Demoralization has also been observed in women with a history of SB [33] and in patients visiting EDs for SB [34]. Therefore, the studies mentioned above indicate a relationship between SB and demoralization; yet, results are heterogeneous and limited in number, urging the need for further research to confirm this relationship and specify its dynamics. In addition, further investigation of additional theoretical issues to specify the interplay between demoralization and SB is required, particularly concerning the previously outlined characteristics of patients presenting in EDs.

First, demoralization has been differentiated conceptually from depression by the absence of anhedonia [20,35,36,37]. Studies performed on cancer patients showed the statistical independence of demoralization from depression, notably regarding the contribution of these two constructs in the variance of SB [27,29]. Other studies reported an overlap of demoralization and depression in patients with somatic disorders [16,21,28,38,39]. As depression has been regularly associated with SB [40], further clarification of the hypothesized distinction between demoralization and depression is essential.

Second, demoralization has been differentiated from one of its constitutive constructs, namely hopelessness [11,17,20,28,41]. Hopelessness refers to three major aspects, namely negative feelings towards the future (affectively toned associations with the future), loss of motivation (motivational associations), and negative expectations (cognitive associations) [42]. Interestingly, hopelessness was found to be an independent and more powerful predictor of SB than depression [43]. The proportion of variance accounted for the hopelessness in the demoralization influence on SB, given that hopelessness is a sub-construct of demoralization, remains unclear for some authors [44]. 

**Figure 1:**
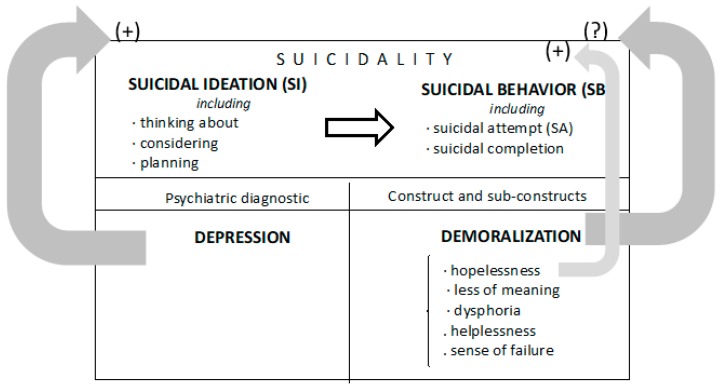
Schematic of the study design to determine the role of demoralization in suicidal patients attending an emergency department (ED). Depression is known to directly influence suicidality (SI + SB). However, less is known about the role of the demoralization construct. The aims of the present study were to: (i) assess the presence and severity of demoralization in suicidal patients visiting an ED; (ii) determine whether the demoralization construct can be parsed independently from depression as a factor associated to suicidality; and (iii) investigate whether the proportion of variance in the relationship between suicidality and demoralization is accounted for by the sub-construct hopelessness.

Accordingly, the discussed literature suggests that further research is required on the validation of demoralization as a specific psychological construct, within the context of its relationship with SI and SA, and its theoretical independence from other constructs, e.g., depression and hopelessness, in playing a central role in SB. The present study assessed the presence and severity of demoralization in patients visiting EDs for SI and SA (Figure 1). Secondly, it examined whether the relationship between demoralization and SI/SA is sustained after controlling for depression, and tested the strength of demoralization as an independent prognostic factor. Finally, it investigated whether the proportion of variance in the relationship between SB and demoralization is accounted for by the sub-construct hopelessness. We hypothesized that: (1) high levels of demoralization are present in suicidal patients visiting EDs; (2) demoralization is a construct that accounts for SI, independently of depression; and (3) the effect of demoralization on SI and SA is independent of the effect of hopelessness on SB.

## 2. Methods

### 2.1. Sample

Adult patients referred to the psychiatric division of the ED of the Geneva University Hospital in Switzerland between October 2014 and February 2016 for SI and/or SA were proposed to participate in the study. Inclusion criteria were the occurrence of SI and/or SA as the reason for transfer to the ED psychiatric ward after initial screening at the general ED, minimum age of 16, informed consent, and completion of the study within 7 days following inclusion time. The nomenclature proposed by Posner mentioned earlier was used to define SI and SA [3]. Evidence that the individual intended to kill him/herself, at least to some degree, can be explicit or inferred from the behavior or circumstance [3]. This study took place as part of a larger research project, approved by the local research ethics committee (project number: 14-168).

Three hundred and sixty-eight patients were approached to participate in the study, with 129 (35.1%) refusing due to various reasons, such as fatigue, poor concentration, lack of interest, insufficient French language level, or healthcare refusal. In addition, 38 individuals were excluded due to non-compliance with the study protocol, the presence of suspicious data, failure to provide consent, second visit to an ED, and failure to return questionnaires within 7 days. A further two participants did not respond to the questionnaires of specific interest, resulting in a final sample of 199 individuals.

### 2.2. Procedure

Participants were requested to fill out a series of questionnaires, which were either administered by the psychiatrist in charge of study inclusion, or self-reported upon ED admission. In the clinician-administered group, participants were questioned on sociodemographic variables, as well as previous history of SA. In addition, participants were administered the Mini-International Neuropsychiatric Interview (MINI) French version 5.0.0 [45], for diagnostic psychiatric screening according to the DSM-IV and ICD-10 criteria. In the self-administered group, participants were requested to complete the Scale for Suicide Ideation (SSI) [46] and the Demoralization Scale (DS), to provide a subjective measurement of current SI and level of current demoralization, respectively. Additional questionnaires not relevant to the current study were also proposed.

### 2.3. Instruments

The SSI [46], which is designed to assess current SI, was proposed to participants in a self-reported version validated in French [47]. The questionnaire comprises 19 items rated on a three-point Likert scale, ranging between 0 and 2 (anchors differ), leading to a maximum score of 38. High scores indicated strong SI and a score of 6 or more has been used as a cut-off for clinically significant ideation in adult patients [48,49]. The SSI addresses various themes associated with SI, such as the desire to live, duration of suicidal thoughts, and the presence of a suicide note. Furthermore, the SSI tends to be zero-inflated, because a score of 0 for items 4 and 5, which are screening items, should automatically result in a total score of 0 [50]. To attenuate this effect, we considered the entire scale, even in participants for whom screening items amounted to 0. Cronbach’s alpha demonstrated the reliability or internal consistency of the sample to be good (*α* = 0.882). 

Demoralization was assessed using the DS [20], whose conceptual theoretical model [18,19] covers five dimensions: loss of meaning, dysphoria, hopelessness, helplessness, and sense of failure. The DS comprises 24 items rated on a five-point Likert scale, ranging from 0 (never) to 4 (all the time). The highest possible score is 96, with a higher score indicating a higher level of demoralization. The DS computes five subscores of loss of meaning (five items), dysphoria (five items), hopelessness (six items), helplessness (four items), and sense of failure (four items). The scale has been developed and validated among cancer and palliative patients in different countries [21,51,52,53,54] with mean scores around 30 [53,55]. In all of the above-mentioned studies, the divergence of the construct of demoralization from depression was shown. In other studies, DS has also been found to detect SI [21,33,34]. The internal consistency in the current sample was excellent (*α* = 0.918).

### 2.4. Statistical Analyses

Descriptive and correlational analyses were performed initially to investigate the relationship between the variables of interest. Bivariate Pearson’s correlations and point-biserial correlations were computed to explore relationships between suicidality and sociodemographic variables. Thereafter, we addressed our first hypothesis in building two regression models. First, hierarchical multiple logistic regression was used to assess the association between demoralization and the fact of being included in the study with SI or with SA. Second, we ran a hierarchical multiple linear regression analysis, to explore the influence of demoralization to SI, as measured by the SSI. In both analyses, we controlled for sociodemographic variables significantly associated with the SSI. Similarly, we included a diagnosis of major depressive episodes in these models, to address our second hypothesis. Finally, we used a similar hierarchical multiple linear regression model to address our third hypothesis in replacing demoralization by hopelessness among the independent variables. Statistical analyses were conducted using Statistica version 13.0 (StatsSoft Inc., Tulsa, OK, USA). A significance level of 0.05 was considered for the interpretation of results.

## 3. Results

### 3.1. Descriptive and Sociodemographic Characteristics

Participants (*N* =199) were predominantly women (60.3%), aged 34.206 ± 14.419 years, and had 0.749 ± 1.058 children (Table 1). The majority of participants were of Swiss citizenship (58.3%), single (65.3%), currently employed (57.8%), and perceived themselves to be of low to middle socioeconomic status (81.9%). Inclusions were mainly based on SI only (65.8%); however, 62.8% of participants had a personal history of SA. In addition, 177 individuals (88.9%) had a past or current psychiatric diagnosis according to the MINI, with a mean 2.156 ± 1.58 diagnoses. The most common diagnosis was current major depressive episode (*n* = 139, 69.8%).

As described in Table 1, the mean SSI score is situated above the threshold of 6, revealing a clinically significant level of SI. The total mean score of the DS indicates a level of demoralization above the one classically described in cancer or end of life patients, the hopelessness subscale showing the highest score, followed by dysphoria, loss of meaning in life, helplessness, and a sense of failure.

Bivariate Pearson’s correlations and point-biserial correlations between variables of interest, i.e., SSI and DS and sociodemographic variables, were performed. The SSI was strongly positively correlated with the DS (*r* = 0.561, *p* < 0.05) and associated with most sociodemographic factors, with correlations of similar valence and amplitude. SSI and DS scores were also positively correlated with the number of psychiatric diagnoses (*r_SSI_* = 0.140, *r_DS_* = 0.230, *p* < 0.05) and current diagnosis of major depressive episode (*r_SSI_* = 0.234, *r_DS_* = 0.210, *p* < 0.05, coding: no diagnosis = 0, presence of a diagnosis = 1), identified by the MINI. SA as an inclusion criteria was negatively correlated with SSI and DS scores (*r_SSI_* = -0.191, *r_DS_* = −0.234, *p* < 0.05, coding: SI = 0, SA = 1). Additionally, SSI and DS scores were also negatively correlated with having children (*r_SSI_* = −0.196, *r_DS_* = −0.192, *p* < 0.05, coding: no children = 0, children = 1). Furthermore, the SSI was negatively associated with a current relationship (*r* = −0.155, *p* < 0.05, coding: single = 0, in a relationship = 1), whereas the DS was negatively correlated with age (*r* = −0.240, *p* < 0.05). A significant relationship was not observed for other association measures.

### 3.2. Demoralization and SI/SA, and Demoralization and Depression

A multiple logistic regression analysis was applied using the study inclusion criterion as a dependent variable. In a first step, we controlled for the sociodemographic and clinical variables that were significantly associated with SI measured by the SSI in the descriptive analyses. These variables were the diagnosis of current major depressive episodes, the number of psychiatric diagnoses, presence of children, and current relationship. According to the regression model, a small proportion of the variance in the study inclusion criterion could be explained by these variables (deviance = 252.157, df = 194, Cox-Snell *R*^2^ = 0.017, Nagelkerke *R*^2^ = 0.024); however, none significantly predicted the study inclusion criterion (Table 2). Subsequently, DS was added in a second step, as a measure of demoralization. A large proportion of the variance in the dependent variable could be accounted for (deviance = 242.724, df = 193, Cox-Snell *R*^2^ = 0.063, Nagelkerke *R*^2^ = 0.087), with the DS score being a significant predictor of SI as an inclusion criterion (OR = 0.969 (CI 95% 0.950 – 0.989), *p* = 0.003).

Next, we designed a multiple linear regression model, with SI measured by the SSI as the dependent variable. In an initial step, identical sociodemographic and clinical variables were considered, as described in the multiple logistic regression model above. This regression model significantly predicted suicidal ideation (*F*(4, 194) = 6.499, *p* < 0.001; Table 3). Current diagnosis of major depressive episode (*b* = 3.861, *t* = 3.054, *p* = 0.003) and participants with fewer children (*b* = −2.816, *t* = −2.485, *p* = 0.014) were predictors of the dependent variable. In a second step, demoralization, as measured by the DS, was added to the model, and was also significantly associated with suicidal ideation (*F*(5, 193) = 20.766, *p* < 0.001). Current diagnosis of major depressive episode (*b* = 2.624, *t* = 2.389, *p* = 0.018) remained a predictor of the dependent variable, as well as higher demoralization scores (*b* = 0.245, *t* = 8.292, *p* < 0.001).

### 3.3. Demoralization and Hopelessness

Finally, we replaced demoralization with hopelessness in the next regression model. Current diagnosis of major depressive episode (*b* = 2.725, *t* = 2.338, *p* = 0.020), absence of children (*b* = −2.127, *t* = −2.049, *p* = 0.042), and a higher hopelessness subscale score of the DS (*b* = 0.663, *t* = 6.372, *p* < 0.001) were identified as determinants of suicidal ideation. This model significantly predicted the dependent variable (*F*(5, 193) = 14.380, *p* < 0.001, adjusted *R^2^* = 0.253).

## 4. Discussion 

The present study investigated the role of demoralization in patients attending a psychiatric ED for SI and SA. Regarding our first hypothesis, demoralization and SI were strongly positively correlated, and a higher demoralization score was associated with SI. To our knowledge, this is the first study investigating the role of demoralization in suicidal patients with various psychiatric diagnoses. Previous studies addressed different populations. Butterworth et al. [30] reported elevated rates of SB in demoralized, disadvantaged individuals after a welfare reform. Community-dwelling older women with a history in the past five years of SB presented with high levels of demoralization, compared to controls without SB [33]. However, in this study, psychological factors, not eventual psychiatric diagnoses, were considered. In patients with schizophrenia, Drake and colleagues [31] first elaborated on a demoralization syndrome, postulating that repeated exacerbation of psychotic symptoms and functional deterioration can lead to suicide, especially in individuals with good premorbid adjustment and insight (for a review of the role of demoralization role in suicide risk in schizophrenia, see [32]). In a cohort of patients attending the ED for SB, the mean demoralization score was almost twice as high as that of patients with cancer [34]. However, the aforementioned study excluded patients having a specific diagnosable psychiatric disorder. 

Interestingly, the DS and SSI were negatively correlated with SA as an inclusion criterion. To our knowledge, no prior studies have examined this factor. The finding that demoralization was not associated with the inclusion of SA could be justified by the fact that demoralization would explain less serious manifestations, such as SI, but not more serious manifestations, such as SA. However, this would not be coherent with the finding that SI is not associated with SA as an inclusion criterion. In our previous study performed on the same sample set, participants visiting EDs for a SA had a lower SI than those with SI only [56]. The findings on SA for both demoralization and SI could be explained by a potential psychological process unfolding after a SA, with mitigating effects on SI. Data on the attitudes and reactions of individuals following attempted suicide are limited. However, the initial recovery of aspects after an attempted suicide has been reported to be related to at least two demoralization subconstructs, namely hopelessness and the loss of meaning in life [57,58].

As for the second hypothesis, demoralization was mildly positively correlated with a diagnosis of a current major depressive episode (0.230). In contrast to a current major depressive episode, demoralization predicted patient inclusion based on SI rather than SA, which did not make a distinction. Both demoralization and current major depressive episodes positively predominated the SI measured dimensionally by the SSI, even by controlling the demoralization or major depressive episode effect. Altogether, our results suggest that demoralization is associated with a major depressive episode. However, demoralization differs from a current major depressive episode and is most likely more sensitive, as shown by linear regression, validating the specificity of demoralization to depression and its role in suicidality. This finding is in line with previous research in patients with cancer, in which demoralization in comorbidity with [28], or independent of, depression [27,29] increased the risk of SB. 

Concerning our third hypothesis, hopelessness positively predicted SI. This result was expected because hopelessness is a constitutive element of demoralization, as measured by the DS. By controlling for the same variables, hopelessness, however, explained a smaller part of the variance in SI than demoralization (25.3% vs. 33.3%). On the one hand, this delineates a dissimilarity between the two constructs; yet, on the other hand, it also suggests that hopelessness accounts for a greater part of the variance in SI than the other subconstructs of demoralization. Yet, due to the non-nested nature of these two models, both constructs could not be compared directly and, therefore, the significance of this difference could not be assessed. In their recent systematic review on the role of demoralization and hopelessness in suicide risk in schizophrenia, Berardelli et al. [32] supported the hypothesis that the association between depression and suicide is moderated by hopelessness.

Potential relationships between demoralization and SI with sociodemographic variables were investigated, which has not yet been previously performed. As reported in our previous studies using the same sample cohort [53,54,55], the characteristics of sociodemographic variables were representative of individuals visiting the ED for a non-lethal suicidal event. Unsurprisingly, demoralization and SI, which were strongly positively correlated, showed a negative association to most sociodemographic factors considered as significant protective factors for SI and SA, such as having a child, being in a relationship, and the presence of less severe psychiatric disorders [59,60]. Demoralization was negatively associated with age. These findings could suggest that demoralization and the correlated SI can be affected by crucial existential conditions, such as childbirth, marriage, the burden of psychiatric diagnoses, and younger age or the transition to a more mature phase in life. 

The present study has several limitations. First, the age distribution of the subjects varies greatly, which is confirmed by the standard deviation of the mean age. Second, regression analyses of cross-sectional data were used; therefore, the findings should be interpreted with caution. Further longitudinal prospective studies, including the trajectory of demoralization over time before and after a significant life event, are required to validate the findings. Third, the lack of a case-control design in this study limits the validity of our conclusions. Fourth, the MINI was used for psychiatric diagnostics and has limited diagnostic accuracy. This is particularly relevant regarding diagnosing depression, which was not based on specific scales, such as the Montgomery-Asberg Depression Rating Scale or the Hamilton Depression Rating Scale. In addition, a specific scale for evaluating hopelessness, such as the Beck Hopelessness Scale, was not used. Fifth, the relationship between demoralization and psychotic disorders was not investigated. Sixth, about 35% of the ED visitors refused to participate in the study. We also lacked information on participants subsequently excluded from the study and, therefore, we were unable to control for possible inclusion biases. Moreover, if demoralization contributed in the refusal of these solicited patients, the power of our findings would be underestimated. Finally, demoralization was tested only in patients attending EDs for SI and SA. This factor foreclosed the potential to explore its validity in patients attending EDs for reasons other than SB, to detect possible silent SI. 

## 5. Conclusions

The present study provided formal support for the association of demoralization with SI. Clinicians should keep in mind that the monitoring of this construct can improve suicidal evaluation, especially in the heterogeneous patient pool of the ED, with particular relevance on patients with an undetected suicide risk. In particular, the assessment of demoralization could represent an instrument in patients that do not present with depression, but may be suicidal. Furthermore, demoralization could represent a clinically useful concept to broaden the comprehension of the suffering of the suicidal patient and a target for psychotherapeutic interventions. Further studies to elucidate the preliminary findings of this study, addressing, in particular, its limitations, are required.

## Figures and Tables

**Table 1:** Psychosocial characteristics and mean scores of scales measuring constructs/sub-constructs

Psychosocial Characteristic (*N* = 199)	Type	*n*	Percentage
Gender	Women	120	60.3
	Men	79	39.7
Age	≤ 50 years	166	83.4
	≥ 50 years	33	16.6
Citizenship	Swiss	116	58.3
	Non-Swiss	83	41.7
Marital status	In a relationship	69	34.7
	Single	130	65.3
Children	Yes	78	39.2
	No	121	60.8
Professional status	Employed/Student	115	57.8
	No activity	84	42.2
Perceived wealth status	High (categories 1–3)	36	18.1
	Low (categories 4–6)	163	81.9
Inclusion criterion	Suicidal ideations	131	65.8
	Suicide attempt	68	34.2
History of suicide attempt	Yes	125	62.8
	No	74	37.2
**Constructs**	**Sub-constructs**	**Mean**	**SD**
Suicidal ideation (SSI)	Total score	15.1	7.6
Demoralization (DS)	Total score	61.1	15.9
	Meaning in life	11.5	4.7
	Hopelessness	17.2	4.7
	Helplessness	11.1	3.4
	Sense of failure	8.5	3.6
	Dysphoria	12.8	4.3

**Table 2:** Hierarchical multiple logistic regression equation, predicting the probability of being included with suicidal ideation vs. suicide attempt (coding values: 0 = suicidal ideation (n = 131), 1 = suicide attempt (n = 68)).

Predictors Entered in Set	Cox-Snell *R*^2^	Nagelkerke *R*^2^	Odds Ratios	Lower CI 95%	Upper CI 95%	*p*
1						
	0.017	0.024				
MINI—major depressive episode			0.667	0.324	1.372	0.271
MINI—number of diagnoses			1.050	0.851	1.296	0.646
Children			1.209	0.634	2.305	0.565
Marital status			1.474	0.763	2.850	0.248
2						
MINI—major depressive episode	0.063	0.087	0.771	0.368	1.618	0.492
MINI—number of diagnoses			1.115	0.899	1.384	0.322
Children			1.031	0.528	2.016	0.928
Marital status			1.337	0.675	2.648	0.405
Demoralization			0.969	0.950	0.989	**0.003**

MINI = Mini-International Neuropsychiatric Interview, French version 5.0.0 [45]. Note: covariates included the MINI diagnosis of major depressive episode, the MINI total number of diagnoses, the number of children and the marital status. In bold, *p*-values significant at the 0.05 threshold.

**Table 3:** Multiple linear regression equation, predicting suicidal ideation as measured by the SSI (n = 199).

Predictors Entered in Set	*F*	df	*R* ^2^	Adjusted *R*^2^	*b*	*t*	*p*
1	6.499	4, 194	0.118	0.100			**0.000**
MINI—major depressive episode					3.861	3.054	**0.003**
MINI—number of diagnoses					0.368	0.827	0.409
Children					−2.816	−2.485	**0.014**
Marital status					−1.937	−1.662	0.098
2	20.766	5, 193	0.350	0.333			**0.000**
MINI—major depressive episode					2.624	2.389	**0.018**
MINI—number of diagnoses					−0.161	−0.499	0.618
Children					−1.480	−1.498	0.136
Marital status					−1.206	−1.198	0.232
Demoralization					0.245	8.292	**0.000**
3	14.380	5, 193	0.271	0.253			
MINI—major depressive episode					2.725	2.338	**0.020**
MINI—number of diagnoses					0.039	0.115	0.909
Children					−2.127	−2.049	**0.042**
Marital status					−1.042	−0.973	0.332
Hopelessness					0.663	6.372	**0.000**

Note: covariates included the MINI diagnosis of major depressive episode, the MINI total number of diagnoses, the number of children and the marital status. In bold, *p*-values significant at the 0.05 threshold.
